# Predictions of CD4 lymphocytes’ count in HIV patients from complete blood count

**DOI:** 10.1186/1756-6649-13-3

**Published:** 2013-09-14

**Authors:** Javier O Rodríguez, Signed E Prieto, Catalina Correa, Carlos E Pérez, Jessica T Mora, Juan Bravo, Yolanda Soracipa, Luisa F Álvarez

**Affiliations:** 1 Insight Group. Research Area and Special Internship “Mathematical and Physical Theories Applied to Medicine”, Medicine Faculty, Universidad Militar Nueva Granada, Cr 11 No.101-80 Bogotá, Colombia; 2 Medicine Faculty, Universidad Militar Nueva Granada, Cr 11 No.101-80 Bogotá, Colombia

**Keywords:** CBC, CD4, HIV, Predictions, Sets theory

## Abstract

**Background:**

HIV diagnosis, prognostic and treatment requires T CD4 lymphocytes’ number from flow cytometry, an expensive technique often not available to people in developing countries. The aim of this work is to apply a previous developed methodology that predicts T CD4 lymphocytes’ value based on total white blood cell (WBC) count and lymphocytes count applying sets theory, from information taken from the Complete Blood Count (CBC).

**Methods:**

Sets theory was used to classify into groups named A, B, C and D the number of leucocytes/mm^3^, lymphocytes/mm^3^, and CD4/μL^3^ subpopulation per flow cytometry of 800 HIV diagnosed patients. Union between sets A and C, and B and D were assessed, and intersection between both unions was described in order to establish the belonging percentage to these sets. Results were classified into eight ranges taken by 1000 leucocytes/mm^3^, calculating the belonging percentage of each range with respect to the whole sample.

**Results:**

Intersection (A ∪ C) ∩ (B ∪ D) showed an effectiveness in the prediction of 81.44% for the range between 4000 and 4999 leukocytes, 91.89% for the range between 3000 and 3999, and 100% for the range below 3000.

**Conclusions:**

Usefulness and clinical applicability of a methodology based on sets theory were confirmed to predict the T CD4 lymphocytes’ value, beginning with WBC and lymphocytes’ count from CBC. This methodology is new, objective, and has lower costs than the flow cytometry which is currently considered as Gold Standard.

## Background

HIV infection has affected around 60 million people to date [1]. In 2009, there were 33.3 million people living with HIV worldwide; 2.6 million new cases were presented and 1.8 million deaths were secondary to AIDS in the same year [2]. By 2009, Sub-Saharan Africa was the leading region in the world for deaths caused by AIDS, recording 1.3 million cases [2]. Even though AIDS is a global problem, countries with fewer resources are mostly affected [2,3].

HIV is a retrovirus that mainly affects T cells and those cells that express CD4, such as macrophages, follicular dendritic cells and lymph nodes [4]. In the natural history of HIV infection, there is an initial decrease in the number of TCD4 lymphocytes that relates to the clinical primary infection (2 weeks after infection); then a partial recovery occurs, due to atypical lymphocytes and to an increase in T CD8 lymphocytes (3–4 weeks after exposure). Finally the number of lymphocytes decreases again; slowly during the latent period and faster during the final stage which is characterized by a notorious immunodeficiency with CD4 counts below 500 CD4/μl^3^[4]. For this reason, both the percentage of T CD4 lymphocytes and the occurrence of opportunistic infections define the stages of HIV infection and provide treatment guidelines. Currently this percentage is one of the referenced biological and immunological markers for HIV infection and AIDS control and it is also a predictor of mortality [5]. Its determination is the result of three laboratory steps: count of WBC, percentage of WBC that are lymphocytes or differential count, and percentage of CD4 lymphocytes. This last stage is performed by a technique known as “immunophenotyping by flow cytometry”, which consists in the detection of CD4 antigenic determinants on the surface of WBC using monoclonal antibodies labeled with fluorescein [6,7]. However, this procedure has several limitations, such as a delay of more than 24 hours between blood collection and its processing, and the costs of equipment and reagents for flow cytometry, which make it inaccessible to some developing countries, especially Africa [5-9].

Given the large impact that HIV/AIDS represent for global public health, it has been sought to make flow cytometry more accessible by implementing simplified flow cytometers that are chargeable by battery or solar panels [8]. On the other hand it has been sought to replace it by methods of CD4 lymphocytes count prediction from CBC parameters [10,11], epidemiological variables [12,13] or machine learning [14]. There is a cross-sectional study of CD4 prediction from CBC parameters, which used the combined values of total T lymphocytes and hemoglobin to deduce CD4 counts <200 cells/μL^3^; however, when this prediction was compared to the deduction based on total lymphocytes, it was found that in male patients sensitivity increased with no changes in specificity, and in female patients sensitivity did not change and specificity decreased [10]. A cross-sectional study that assessed the usefulness of total lymphocyte count as surrogate marker of T CD4 lymphocyte’s count in HIV-positive patients found that there is a high correlation [11]. However, low sensitivity of total lymphocyte count was found in the classification of patients with CD4 counts <200 cells/μL [11]. Another epidemiological study, sought to predict the variability of T CD4 lymphocytes’ decrease in seropositive patients by determining the distribution of CD4 counts in seronegative patients and survival rates after acquiring HIV infection [12]. This model was applied to different populations and individuals showing accuracy predictions over 75% with respect to the real value of T CD4 lymphocytes variability [13]. In the model proposed by Singh and Mars, based on machine-learning, the CD4 final count is obtained from the viral load values and the number of weeks after the first T CD4 lymphocytes’ count, with an accuracy of 83% with respect to the real value [14].

In a previous study, Rodríguez et al. [15] developed a new methodology applying sets theory to predict T CD4 lymphocytes’ count based on individual values of total WBC and lymphocytes obtained from CBCs. In that work 110 CBCs were analyzed and then classified into four sets named A, B, C and D, where union between sets A and C and union between sets B and D were evaluated, as well as the intersection of both unions. These results were classified into eight ranges of 1,000 leukocytes/mm^3^ each, for its evaluation. The conclusion was that ranges below 5000–4000 leukocytes/ml^3^ predict CD4 counts lower than 570 CD4/μL^3^ with effectiveness percentages between 90-100% [15]. This showed that the study of the variation process of T CD4 lymphocytes’ count reveals an underlying mathematical order when observed through theoretical abstractions; this order allows making simple predictions that are independent of virus characteristics or patient variables.

The aim of this work is to validate the clinical application of the methodology developed based on sets theory, applying it to a larger sample of HIV-positive cases.

## Methods

### Definitions

*Determined Sets for the study of leukocytes/mm*^*3*^*, lymphocytes/mm*^*3*^*and CD4/μL*^*3*^*populations*[15]*:*

A. {(x,y,z)/x > =6.800 ^ y > =1.800}

B. {(x,y,z)/x > =6.800 ^ z > =300}

C. {(x,y,z)/x < 6.800 ^ y < =2.600}

D. {(x,y,z)/x < 6.800 ^ z < =570}

Where (x, y, z) is a triplet of values, being “x” the number of WBC, “y” the number of lymphocytes and “z” the T CD4 lymphocytes’ count.

It is a study in which a physical–mathematical previously developed methodology based on sets theory is applied in order to predict T-CD4 lymphocytes’ count. It is based on the mathematical analysis of the total WBC and lymphocytes’ count in HIV-positive patients.

### Sample

Printed CBCs of 800 HIV diagnosed patients were used, without distinction of gender, age, population kind, or clinical variables such as infection stage, hemoglobin value or medications used. The CBCs were taken from storage tests in a physical data-base of the infectologist who participated in the study.

### Procedure

First, records of leukocytes/mm^3^, lymphocytes/mm^3^ and CD4/μL^3^ subpopulation counts measured by flow cytometry were taken. Then, they were organized in descending order according to the WBC number, establishing ranges of 1000 leukocytes/mm^3^. Values higher than 10.000/mm^3^ were assigned to a single range as well as values lower than 3.000/mm^3^, so a total of 9 ranges were established in order to observe mathematical relationships between populations, independent of time or patient’s evolution.

According to the previously developed methodology, records were evaluated by establishing if they belonged or not to sets A∪C and B∪D, as well as to set (A∪C) ∩ (B∪D), using a software that was previously developed based on sets algebra [15]. This software calculates the range of values in which the T CD4 lymphocytes’ count is, beginning with WBC and lymphocytes number from CBC and applying the evaluated predictive methodology.

Results for the 9 leukocytes ranges were assessed, determining the elements number that belong to each set in each range and the percentage of success to which it corresponds to, according to the total number found for each range. In addition, the same values were established for the whole sample. In this work, the belonging percentage of each range to each one of sets is equivalent to the effectiveness percentage of prediction for such range. When a triplet of values belongs to all sets, this fact means that this triplet met with the condition of have a leukocyte value equal to or higher than 6800/mm^3^, with a lymphocyte value equal to or higher than 1800/mm^3^ and with a CD4 cell value equal to or higher than 300/mm^3^ or it may have a leukocyte value lesser than 6800/mm^3^, with a lymphocyte value equal to or lesser than 2600/mm^3^ and with a CD4 cell value equal to or lesser than 570/mm^3^.

### Statistical analysis

Some performance measures were established for each range through a binary classification performance measurement, where True positive (TP) is the number of cases with a correct prediction in the range with respect to real values, False negative (FN) is the number of wrong predictions in the range with respect to real values, and finally True negative (TN) is the total number of correct predictions in the other ranges. The performance measures calculated for each range were Sensitivity (SENS), and Negative Predictive Value (NPV); the first one which was calculated with the next equation:

$$\mathrm{SENS}=\mathrm{TP}/\left(\mathrm{TP}+\mathrm{FN}\right)$$

Otherwise, Negative Predictive Value (NPV) and was calculated by means of the next equation:

$$\mathrm{NPV}=\mathrm{TN}/\left(\mathrm{TN}+\mathrm{FN}\right)$$

### Ethic aspects

This study follows the laws established on articles 11 and 13 of the 008430 Colombia’s Health Ministry resolution of 1993 given that physical calculations were made based on results of medically prescribed tests of the clinical practice, from an anonymous database retrospectively evaluated, with no risks to patients, protecting the integrity and anonymity of participants and with no need of informed consents. The approval of an ethics committee of a specific institution is not needed because it was accessed only numerical values of the database (without access to the names, data source or clinic history of patients), collected specifically for research purposes by one of the authors.

## Results

Belonging of leukocytes, lymphocytes and CD4 cells values to each set in 27 specific samples is shown at the Table 1. In this table, few examples are shown of each range and with different belonging states to the defined sets. Namely, particular triplets of values are shown which do not belong to all the sets, such as the triplet 6850; 1140; 345 which only belong to the B∪D set and as a consequence it cannot belong to the intersection set (A ∪ C) ∩ (B ∪ D) . Instead, several triplets of values are shown which belong to all sets, such as the triplet 2880; 1150; 54 (See Table 1).

**Table 1:** Application of the proposed methodology to 27 specific samples

**Total leukocytes**	**Total lymphocytes**	**CD4**	**A**∪**C**	**B**∪**D**	**(A**∪**C)** ∩ **(B**∪**D)**
17850	2200	223	X		
13180	2160	362	X	X	X
10000	2480	509	X	X	X
9920	5060	375	X	X	X
9570	2960	602	X	X	X
9000	1920	254	X		
8990	3440	472	X	X	X
8050	850	163			
8020	3320	346	X	X	X
7940	2370	461	X	X	X
7539	3230	444	X	X	X
7070	1910	652	X	X	X
6960	3710	522	X	X	X
6850	1140	345		X	
6000	1290	421	X	X	X
5870	1080	134	X	X	X
5600	1770	247	X	X	X
5200	1800	359	X	X	X
4999	1680	522	X	X	X
4510	1570	433	X	X	X
4070	1680	645	X		
3860	1410	396	X	X	X
3380	1650	20	X	X	X
3010	1570	109	X	X	X
2880	1150	54	X	X	X
2160	1240	110	X	X	X
1200	684	44	X	X	X

“X” represents the sample belonging to each assessed set.

Table 2 shows that effectiveness percentage of the prediction for set A ∪ C according to each range, was between 68.42% and 100%, for set B ∪ D was between 65.66% and 100%, and for intersection set (A ∪ C) ∩ (B ∪ D) was between 55.64% and 100%. Effectiveness percentage of the prediction for the total number of cases to set A ∪ C was 81%, and to set B∪D was 80%, whereas for total number of cases to intersection set (A ∪ C) ∩ (B ∪ D) was 73.25% (See Table 2), being equal or above 73.91% in 6 out of the 9 established ranges, and over 81.44% in 5 ranges. This effectiveness percentage to the intersection (A ∪ C) ∩ (B ∪ D) was higher for the upper and lower ranges; which was between 83.05% and 83.33% for the ranges of 8000–8999 and 9000–9999, respectively; and was between 81.44% and 91.89% for the ranges of 4000–4999 and 3000–3999, respectively. For the range of leukocytes below 3000, that has more utility in clinical setting, the effectiveness percentage was of 100% (See Table 2).

**Table 2:** Elements number (No.) that conform the established sets according to each range and effectiveness percentage of the prediction (%) according to sets theory

**Ranges of leukocytes**	**Cases number per range**	**A**∪**C**		**B**∪**D**		**(A**∪**C)** ∩ **(B**∪**D)**	
		**No.**	**%**	**No.**	**%**	**No.**	**%**
10000 or more	23	22	95.65	18	78.26	17	73.91
9999 to 9000	42	38	90.48	36	85.71	35	83.33
8999 to 8000	59	56	94.92	48	81.36	49	83.05
7999 to 7000	99	90	90.91	65	65.66	61	61.62
6999 to 6000	133	91	68.42	97	72.93	74	55.64
5999 to 5000	186	159	85.48	149	80.11	129	69.35
4999 to 4000	167	161	96.41	142	85.03	136	81.44
3999 to 3000	74	73	98.65	68	91.89	68	91.89
2999 or less	17	17	100	17	100	17	100
**Total**	800	707	88	640	80	586	73.25

### Statistical analysis results

TP values ranged between 17 and 136, TN values were between 450 and 569, and FN between 0 and 59. Values for SENS ranged between 0.56 and 1, and values for NPV were between 0.89 and 1. The highest SENS values were for the ranges of 10000 leukocytes or more, between 9999–9000, 3999–3000, and for the range of 2999 leukocytes or less; the first three had values of 0.99 and the last one of 1.The NPVs showed values equal to or greater than 0.98 in 5 out of the 9 assessed ranges (See Table 3).

**Table 3:** Values for the statistical analysis made

**Ranges of leukocytes**	**TP**	**TN**	**FN**	**SENS**	**NVP**
10000 or more	17	569	6	0.74	0.99
9999 to 9000	35	551	7	0.83	0.99
8999 to 8000	49	537	10	0.83	0.98
7999 to 7000	61	525	38	0.62	0.93
6999 to 6000	74	512	59	0.56	0.90
5999 to 5000	129	457	57	0.69	0.89
4999 to 4000	136	450	31	0.81	0.94
3999 to 3000	68	518	6	0.92	0.99
2999 or less	17	569	0	1.00	1.00

## Discussion

This is the first work in which a new predictive methodology of T CD4 lymphocytes’ count is applied to a sample of 800 HIV-positive patients. This methodology was developed beginning with the analysis of WBC and lymphocytes’ count from CBC, and it is based on sets theory. Its predictive percentages are equal or above 73.91% for 6 out of 9 measured ranges, confirming its predictive capacity and clinical applicability independently of epidemiological and clinical variables. Sensitivity values over 0.80 were founding 5 of the 9 measured ranges; specificity was not calculated, given that there are no False Positives.

Taking into account that the starting of anti-retroviral treatment is suggested at 300 CD4 cells/cm^3^, this predictive methodology showed an effectiveness percentage of prediction of 100% when leukocyte values were less than 3000.This means that a value of CD4 less than 570/mm^3^ is predicted for all these cases.

The belonging percentage to set A ∪ C is greater than the percentage to set B ∪ D, showing the specificity of T CD4 lymphocytes’ values and evidencing the difficulty to find results that allow their prediction. In contradistinction to the previous work [15], one more range of leukocytes, from 3999 to 3000, was quantified in this work in order to study more specifically the ranges of values that have greater clinical importance. High values in the predictions were found, with percentages over 73% and even of 100% for high and low ranges, which are clinically the most important.

The mathematical theory through which predictions are obtained does not allow the establishment of False Positives in the statistical analysis, given that each set, as well as the intersections that constitute the prediction, exclude the possibility of finding triplets that allow obtaining a False Positive prediction. This is the reason why it is not possible to establish a positive predictive value, showing that this mathematical inductive way of thinking can’t be taken directly from traditional statistical parameters; instead of that, the sets algebra way of thinking achieves deductive predictions of clinical importance.

Works performed with the aim to simplify and reduce costs of HIV patients follow-up are mostly epidemiological, with limitations in the prediction of immunological biomarkers [10-13], given that its study from epidemiological variables or virus characteristics does not allow a complete, rigorous, objective, and also simple and reproducible analysis of the immunological response of such patients. Such is the case of cross-sectional descriptive studies that try to deduce T CD4 lymphocytes from CBC parameters such as total lymphocytes’ count or hemoglobin. However, although a correlation between these parameters has been found, the sensitivity of deductions varies according to gender [10] or to CD4 count itself [11]. Also, studies have sought to predict the variability of T CD4 lymphocytes’ decrease in seropositive patients by determining the distribution of CD4 counts in seronegative patients and survival rates after acquiring HIV infection [12,13]. Other studies based on machine learning, as the one proposed by Singh and Mars [14] to obtain the latest CD4 count have an accuracy not greater than 90% and require a previous count of T CD4 lymphocytes and values of viral load, which represent a moderate additional cost.

Based on neural networks and machine learning, some methodologies propose viral load measurement as marker of treatment response in HIV-infected patients. The limitation of these experimental methodologies is that they don’t take into account the immune response of the patient, but genotypic virus characteristics [16-19], which result in expensive flow cytometry tests [8,9]. In contrast to these studies, the present study applies a methodology that uses more accessible data such as the CBC and analyses it objectively from a sets theory approach in order to deduce the value of T CD4 lymphocytes with a high success percentage. This study provides useful scientific contributions for the development of control measures and management of HIV/AIDS pandemic; contributions of clinical applicability that may optimize care and follow-up of patients that suffer from this disease.

In the context of dynamic systems, a descriptive but not predictive model of the immune response to HIV dynamics was developed by plotting how T CD4, CD8, B lymphocytes and antibodies act, and how the viral load progresses [20]. The present paper makes an analysis of the variation process of WBC and lymphocytes populations in HIV patients, but also allows the prediction of CD4 subtype. Furthermore, since it is based on a mathematical approach, it does not require statistical analysis, as it is not required in the study of physical phenomena such as predicting the trajectory of planets or an eclipse.

Likewise in this work, where predictions are achieved from mathematical theories, in different areas of medicine other phenomena have also characterized and predicted from several mathematical and physical theories, evidencing the mathematical self-organizations of these phenomena [21-30].

## Conclusions

This study confirms the predictive capacity of the developed methodology based on sets theory to determine the number of T CD4 cells based on WBC and lymphocytes’ count, achieving a 91.89% effectiveness for the range between 3000 and 3999 leukocytes, and 100% for the range below 3000 leukocytes. This methodology can be useful to determine the number of CD4 in places where there is no easy access to flow cytometry, reducing costs in determining the state of patients with HIV/AIDS.

## Competing interests

The authors declare that they have no competing interests.

## Authors’ contributions

JOR developed the original idea and coordinated the study, SEP and CC made the data analysis and contributed in drafting and revising the manuscript; CEP revised the manuscript and made data recollection and its systematization; JTM and JB revised the manuscript and participated in data recollection and its systematization; YS and LA contributed in drafting and revising the manuscript. All authors read and approved the final manuscript.

## Pre-publication history

The pre-publication history for this paper can be accessed here:

http://www.biomedcentral.com/1756-6649/13/3/prepub

## Dedication

The authors dedicate this work to their children and to all HIV patients in the world. To Mariana Pajón: for the joy that you gave to Colombia.

## References

[B1] Instituto Nacional de Salud Subdirección de Vigilancia y Control en Salud Pública 2010 Bogotá: Informe Epidemiológico Nacional 2009 (Colombia)

[B2] WHO, UNAIDS UNAIDS Report on the Global AIDS epidemic 2010 Joint United Nations Programme on HIV/AIDShttp://www.unaids.org/en/media/unaids/contentassets/documents/unaidspublication/2010/20101123_globalreport_en.pdf

[B3] Sabogal A Grupo ITS Informe de VIH-SIDA Colombia periodo XIII año 2009. (Colombia) 2010 Bogotá: Instituto Nacional de Salud: Subdirección de Vigilancia y Control en Salud Pública

[B4] Streicher HZ Reitz MS Jr Gallo RC Mandell GL, Bennett JE, Dolin R Human Inmunodeficiency viruses Principles and Practice of Infectious Diseases 2000 New York: Churchill Livingstone 1874 87

[B5] Brown E Otieno P Mbori-Ngacha D Farquhar C Obimbo E Nduati R Overbaugh J John-Stewart GC Comparison of CD4 Cell Count, Viral Load, and Other Markers for the Prediction of Mortality among HIV-1–Infected J Infect Dis 2009 199 9 1292 1300 10.1086/597617 19317628 PMC2758232

[B6] Centers for Disease Control and Prevention 1997 Revised Guidelines for Performing CD4+ T-Cell Determinations in Persons Infected with Human Immunodeficiency Virushttp://wwwn.cdc.gov/mpep/pdf/tli/rr4602.pdf 9011774

[B7] Mandy F Nicholson J McDougal JS Guidelines for Performing Single-Platform Absolute CD4+ T-Cell Determinations with CD45 Gating for Persons Infected with Human imunodeficiency Virushttp://www.cdc.gov/mmwr/preview/mmwrhtml/rr5202a1.htm 12583540

[B8] Zijenah L Kadzirange G Madzime S Borok M Mudiwa C Tobaiwa O Mucheche M Rusakaniko S Katzenstein DA Affordable flow cytometry for enumeration of absolute CD4+ T-lymphocytes to identify subtype C HIV-1 infected adults requiring antiretroviral therapy (ART) and monitoring response to ART in a resource-limited setting J Transl Med 2006 4 33 10.1186/1479-5876-4-33 16907973 PMC1586214

[B9] Imade GI Badung B Pam S Agbaji O Egah D Sagay AS Sankalé JL Kapiga S Idoko J Kanki P Comparison of a new, affordable flow cytometric method and the manual magnetic bead technique technique for CD4 T-lymphocyte countining in a Northern Nigeria Setting Clin Diag Lab Immunol 2005 12 224 227 10.1128/CDLI.12.1.224-227.2005 PMC540213 15643012

[B10] Budiono W Total lymphocyte count and hemoglobin combined to predict CD4 lymphocyte counts of less than 200 cells/mm(3) in HIV/AIDS Acta Med Indones 2008 40 2 59 62 19054881

[B11] Gitura B Joshi MD Lule GN Anzala O Total lymphocyte count as a surrogate marker for CD4+ t cell count in initiating antiretroviral therapy at Kenyatta National Hospital Nairobi East Afr Med J 2007 84 10 466 72 10.4314/eamj.v84i10.9564 18232267

[B12] Williams BG Korenromp EL Gouws E Schmid GP Auvert B Dye C HIV Infection, Antiretroviral Therapy, and CD4+ Cell Count Distributions in African Populations J Infect Dis 2006 194 1450 8 10.1086/508206 17054076

[B13] Williams BG Korenromp EL Gouws E Dye C The rate of decline of CD4 T-cells in people infected with HIVhttp://arxiv.org/ftp/arxiv/papers/0908/0908.1556.pdf

[B14] Singh Y Mars M Support vector machines to forecast changes in CD4 count of HIV-1 positive patients Sci Res Essay 2010 5 17 2384 2390

[B15] Rodríguez J Prieto S Bernal P Pérez C Correa C Vitery S Teoría de conjuntos aplicada a poblaciones de leucocitos, linfocitos y CD4 de pacientes con VIH. Predicción de linfocitos T CD4, de aplicación clínica Rev Fac Med 2011 19 2 148 156

[B16] Larder B Wang D Revell A Montaner J Harrigan R De Wolf F Lange J Wegner S Ruiz L Pérez-Elías MJ Emery S Gatell J Monforte AD Torti C Zazzi M Lane C The development of artificial neural networks to predict virological response to combination HIV therapy Antivir Ther 2007 12 1 15 24 17503743

[B17] Altman A Däumer M Beerenwinkel N Peres Y Schülter E Büch J Predicting the Response to Combination Antiretroviral Therapy: Retrospective Validation of geno2pheno-THEO on a Large Clinical Database JID 2009 199 999 1006 10.1086/597305 19239365

[B18] Altmann A Rosen-Zvi M Prosperi M Aharoni E Neuvirth N Schülter E Büch J Struck D Peres Y Incardona F Sönnerborg A Kaiser R Zazzi M Lengauer T Comparison of Classifier Fusion Methods for Predicting Response to Anti HIV-1 Therapy PLoS ONE 2008 3 10 e3470 10.1371/journal.pone.0003470 18941628 PMC2565127

[B19] Wang D DeGruttola V Hammer S Harrigan R Larder B Wegner S Winslow D Zazzi M A Collaborative HIV Resistance Response Database Initiative: Predicting Virological Response Using Neural Network Models (Poster presentation) 2002 Seville: A Collaborative HIV Resistance Response Database Initiative: Predicting Virological Response Using Neural Network Models (Poster presentation). XI International HIV Drug Resistance Workshop http://www.hivrdi.org/abstract_3.htm

[B20] Vélez N Torrealdea J Modelado en dinámica de sistemas de la respuesta inmune ante la infección del VIH-1 Ini Inv 2006 1 1 9

[B21] Rodríguez J Diferenciación matemática de péptidos de alta unión de MSP-1 mediante la aplicación de la teoría de conjuntos Inmunología 2008 27 2 63 6810631083

[B22] Rodríguez J Teoría de conjuntos aplicada a la caracterización matemática de unión de péptidos al HLA clase II Rev Cienc Salud 2008 6 1 9 15

[B23] Rodríguez J Teoría de unión al HLA clase II teorías de Probabilidad Combinatoria y Entropía aplicadas a secuencias peptídicas Inmunología 2008 27 4 151 16610631083

[B24] Rodríguez J Bernal P Prieto S Correa C Teoría de péptidos de alta unión de malaria al glóbulo rojo. Predicciones teóricas de nuevos péptidos de unión y mutaciones teóricas predictivas de aminoácidos críticos Inmunología 2010 29 1 7 1910631083

[B25] Rodríguez J Entropía Proporcional De Los Sistemas Dinámicos Cardiacos: Predicciones físicas y matemáticas de la dinámica cardiaca de aplicación clínica Rev Colomb Cardiol 2010 17 115 129

[B26] Rodríguez J Prieto S Melo M Domínguez D Correa C Soracipa Y Forero M Seoane R Tapia D Ramírez S Entropía proporcional de la dinámica cardiaca aplicada al diagnóstico de pacientes de la Unidad de Cuidados Intensivos Medicina 2013 35 100 17 28

[B27] Rodríguez J Mathematical law of chaotic cardiac dynamic: Predictions of clinic application J Med Med Sci 2011 2 8 1050 1059

[B28] Rodriguez J Prieto S Correa C Bernal P Puerta G Vitery S Soracipa Y Muñoz D Theoretical generalization of normal and sick coronary arteries with fractal dimensions and the arterial intrinsic mathematical harmony BMC Med Phys 2010 10 1 6 20846449 10.1186/1756-6649-10-1 PMC2954867

[B29] Rodríguez J Prieto S Correa C Posso H Bernal P Puerta G Vitery S Rojas I Generalización Fractal de Células Preneoplásicas y Cancerígenas del Epitelio Escamoso Cervical. Una Nueva Metodología de Aplicación Clínica Rev Fac Med 2010 18 2 173 181

[B30] Rodríguez J Método para la predicción de la dinámica temporal de la malaria en los municipios de Colombia Rev Panam Salud Publica 2010 27 3 211 218 10.1590/S1020-49892010000300008 20414510

